# Validation of the German Registry for Acute Aortic Dissection Type A Score in predicting 30-day mortality after type A aortic dissection surgery

**DOI:** 10.1093/ejcts/ezad141

**Published:** 2023-04-07

**Authors:** Marco Gemelli, Ettorino Di Tommaso, Roberto Natali, Lauren Kari Dixon, Eltayeb Mohamed Ahmed, Cha Rajakaruna, Vito D Bruno

**Affiliations:** Cardiothoracic Surgery, Bristol Heart Institute, University Hospitals of Bristol and Weston NHS Foundation Trust, Bristol, UK; Department of Cardiac, Thoracic, Vascular and Public Health Sciences, University of Padova, Padova, Italy; Cardiothoracic Surgery, Bristol Heart Institute, University Hospitals of Bristol and Weston NHS Foundation Trust, Bristol, UK; Cardiothoracic Surgery, Bristol Heart Institute, University Hospitals of Bristol and Weston NHS Foundation Trust, Bristol, UK; Cardiothoracic Surgery, Bristol Heart Institute, University Hospitals of Bristol and Weston NHS Foundation Trust, Bristol, UK; Cardiothoracic Surgery, Bristol Heart Institute, University Hospitals of Bristol and Weston NHS Foundation Trust, Bristol, UK; Cardiothoracic Surgery, Bristol Heart Institute, University Hospitals of Bristol and Weston NHS Foundation Trust, Bristol, UK; Cardiothoracic Surgery, Bristol Heart Institute, University Hospitals of Bristol and Weston NHS Foundation Trust, Bristol, UK; Cardiovascular Translational Health Sciences, University of Bristol, Bristol, UK

**Keywords:** Aortic dissection, German Registry of Acute Type A Dissection, European System for Cardiac Operative Risk Evaluation, Risk prediction, Malperfusion

## Abstract

**OBJECTIVES:**

No reliable scores are available to predict mortality following surgery for type A acute aortic dissection (TAAAD). Recently, the German Registry of Acute Aortic Dissection Type A (GERAADA) score has been developed. We aim to compare how the GERAADA score performs in predicting operative mortality for TAAAD to the European System for Cardiac Operative Risk Evaluation (EuroSCORE) II.

**METHODS:**

We calculated the GERAADA score and EuroSCORE II in patients who underwent TAAAD repair at the Bristol Heart Institute. As there are no precise criteria to calculate the GERAADA score, we used 2 methods: a Clinical-GERAADA score, which evaluated malperfusion with clinical and radiological evidence, and a Radiological-GERAADA score, where malperfusion was assessed by computed tomography scan alone.

**RESULTS:**

207 consecutive patients had surgery for TAAAD, and the observed 30-day mortality was 15%. The Clinical-GERAADA score showed the strongest discriminative power with an area under the curve (AUC) of 0.80 [95% confidence interval (CI) 0.71–0.89], while the Radiological-GERAADA score had an AUC of 0.77 (95% CI 0.67–0.87). EuroSCORE II showed acceptable discriminative power with an AUC of 0.77 (95% CI 0.67–0.87).

**CONCLUSIONS:**

Clinical GERAADA score performed better than the other scores and it is specific and easy to use in the context of a TAAAD. Further validation of the new criteria for malperfusion is needed.

**Figure:**
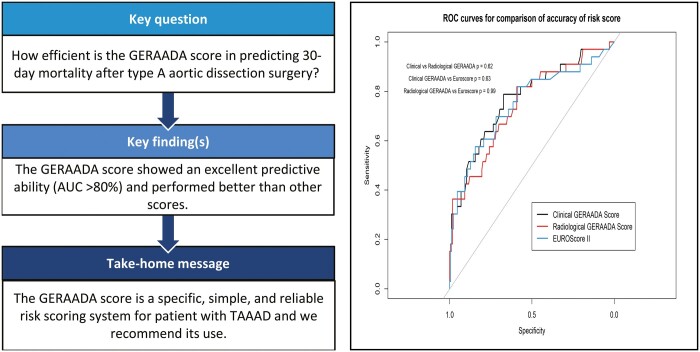

## INTRODUCTION

Type A acute aortic dissection (TAAAD) is a life-threatening condition, with a very high mortality in untreated patients [1]. The surgical repair of the aorta is currently the gold standard for the treatment of TAAAD; the operative mortality has decreased over the years [2, 3] but it is still high, ranging between 9% and 21%. Several risk factors have been correlated to postoperative mortality [4, 5] but a good scoring system to predict the perioperative risk of these patients still needs to be developed. The Society of Thoracic Surgery score and the European System for Cardiac Operative Risk Evaluation (EuroSCORE) II are the most common scoring systems in cardiac surgery, although suboptimal in this population. The Society of Thoracic Surgery score does not consider the surgery of the thoracic aorta and the EuroSCORE II does not consider aortic dissection as an option. New risk scores have been specifically designed to predict outcomes after TAAAD surgery and many preoperative characteristics have been considered as possible predicting factors [6, 7]. Notably, the Penn Classification [8], which stratify patients according to the degree of malperfusion at presentation, represents a reliable preoperative risk assessment tool in TAAAD [8–10]. More recently, the German Registry of Acute Type A Dissection (GERAADA) score, which is based on the degree of malperfusion and the anatomical characteristics of the disease, has been developed and externally validated [3, 11]. Despite the potential benefits of these risk scoring systems, there is no univocal opinion on the best predictive tool for TAAAD surgery. We therefore aim to evaluate the performance of the GERAADA score and EuroSCORE in predicting postoperative mortality after TAAAD surgery.

## MATERIALS AND METHODS

### Ethics statement

The Bristol and Weston University Hospitals NHS Foundation Trust Clinical Audit Review Board approved this retrospective study and waived the need of informed consent from each patient.

### Population

Data for adult patients (>18 years) who had surgery for TAAAD were extracted from the Bristol Heart Institute institutional database. Variables and final calculation of EuroSCORE II were available for all the patients; all data required for calculation of GERAADA score were available in the institutional database. Patients were also categorized according to the University of Pennsylvania Classification. Preoperative computed tomography (CT) scan was available for all the population, except 1 patient. CT scan data were collected retrospectively reviewing official reports and images, which were re-assessed by experienced surgeons and radiologists.

### Definitions

Type A aortic dissection (TAAAD), according to the Stanford Classification, includes all the aortic dissection with an involvement of the ascending aorta with or without the involvement of the aortic arch (and the supra-aortic vessels) and the descending aorta. We define ‘acute’ as any aortic dissection in the first 14 days since the beginning of the symptoms [12].

The EuroSCORE II variables have been considered according to the detailed definitions included in the official website for the calculation of the score (http://www.euroscore.org/calc.html). A precise definition and inclusion/exclusion criteria for the GERAADA score are not present on the website and in the original paper (https://www.dgthg.de/de/GERAADA_Score). One of the criticisms of the GERAADA score is related to the unclear definition of the different types of malperfusion as precise definitions of coronary, visceral and peripheral malperfusion are not available [13]. For these reasons, we have introduced the concepts of clinical and radiological GERAADA score and the second aim of our study is to compare these 2 different ways of calculating it. We have calculated 2 different GERAADA scores: a ‘Radiological’ score, where we evaluate the malperfusion only basis on signs of organs malperfusion at the CT scan, and a ‘Clinical’ score, where we considered clinical signs and symptoms of malperfusion associated with the radiological evidence.

**Figure 1:**
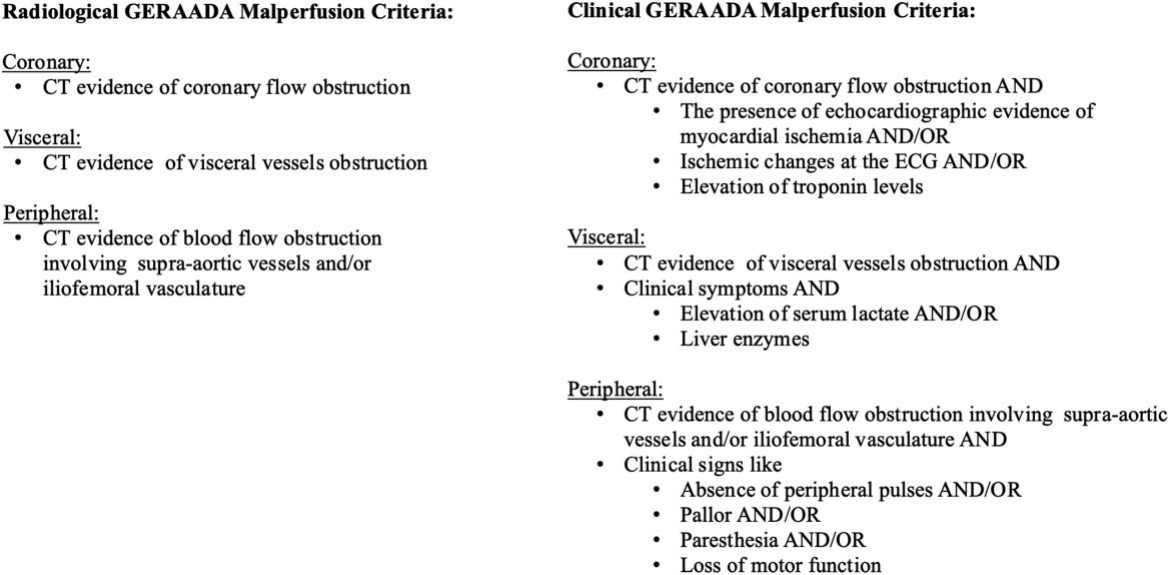
Summary of our criteria for the definitions of malperfusion used in Radiological and Clinical GERAADA Score. CT: computed tomography. ECG: electrocardiography

For the ‘Clinical’ GERAADA score, we considered clinical history, physical examination, CT scan findings and laboratory data. Evidence of malperfusion at the CT scan was defined as an obstruction of >50% of the lumen of the target vessel or significative reduction in contrast enhancement of the related organs [14]. The coronary malperfusion has been defined according to CT scan evidence of malperfusion of the coronary flow and the presence of echocardiographic evidence of myocardial ischaemia and/or ischaemic changes at the electrocardiogram and/or elevation of troponin levels. The visceral malperfusion has been defined as CT scan signs of malperfusion of the abdominal organs blood flow associated with clinical symptoms and elevation of serum lactate and/or liver enzymes. For the peripheral malperfusion, we considered any evidence on the CT scan of malperfusion in the supra-aortic vessels and/or in external iliac or femoral vessels associated with clinical signs like absence of peripheral pulses and/or pallor and/or paresthaesia and/or loss of motor function. For the ‘Radiological’ GERAADA score, we have only considered the CT scan findings. Criteria are summarized in Figure 1.

The University of Pennsylvania Classification, or Penn Classification, is a four-grade classification that categorizes patients based on burden of malperfusion and ischaemia. It is not a numerical risk scoring system like GERAADA score or EuroSCORE, but it is a valuable clinical classification to evaluate quickly TAAAD patients. The patients were classified in class A if they were haemodynamically stable without signs or symptoms of ischaemia. Patients with signs or symptoms of local or generalized malperfusion were divided in the other 3 classes: class B if they were haemodynamically stable but with evidence of branch vessel malperfusion and signs or symptoms of ischaemia; class C if they had global malperfusion from cardiocirculatory collapse; class BC if both characteristics of classes B and C were present [8, 9, 15, 16].

### Operative techniques

All the patients were approached with a median sternotomy; cannulation site was decided basis on the extension of the dissection at the CT scan and according to surgeon’s preference. In most of our patients, we used an axillary cannulation with unilateral or bilateral selective anterograde cerebral perfusion at 24–26°C. All the patients had a replacement of the ascending aorta and we have routinely used an entry tear-oriented approach. If the tear was in the arch we performed, according to the anatomy, an hemiarch or total arch replacement. Frozen elephant trunk was also used in this case, but this procedure was mandatory if further tears were seen distal to the left subclavian artery. Basis on the preoperative echocardiogram and on the intraoperative findings, the surgeon decided if the aortic valve and the aortic root needed to be repaired or replaced.

### Outcomes

The primary end point of the study is 30-day mortality, defined as death by any cause within 30 days from the primary surgery. We calculated Clinical and Radiological GERAADA score, EuroSCORE II and Penn Classification for every patient included and we aimed to evaluate the predictive ability of the Clinical and Radiological GERAADA score and the EuroSCORE II on this outcome. We excluded Penn Classification from the comparison as it is only a classification and does not provide a numerical risk of mortality. A second end point was operative mortality defined as all-cause mortality during the admission in hospital. All the patients who died in the first 30 days after surgery were still in hospital. Other short-term outcomes were cerebrovascular accident, defined on the basis of a focal or global neurological impairment at a physical examination or at CT scan/magnetic resonance imaging, postoperative acute kidney injury, deep sternal wound infection (defined as a surgical site‐related infection affecting the median sternotomy wound and requiring antibiotics and/or surgical management) and in‐hospital length of stay.

### Statistical analysis

Data are reported as mean and standard deviation (in bracket) for continuous numerical variables that were normally distributed and also with median and interquartile range (IQR) (in bracket) for the variables that were not normally distributed and as count and percentages for categorical variables. Normality was assessed with Shapiro–Wilk test. Univariable logistic regression models were run with each scoring system as predictor and mortality as outcome. Receiver operating characteristic curves were drawn to evaluate the diagnostic ability of each score in predicting mortality and the true and false positive rates. Area under the curve (AUC) was used as an aggregate measure of performance and optimal cut-off points for binary classification were established using the Youden’s Index (defined as J = sensitivity + specificity – 1) metric. The AUC is a measurement of the discriminative power of a test and an AUC of 0.5 (50%) suggests no discrimination. An AUC of 0.7–0.8 is considered acceptable, 0.8–0.9 excellent and above 0.9 outstanding [17]. To further evaluate the prediction model for each score calibration plots, decision curve analysis was conducted (Supplementary Material). Comparative analysis between 2 receiver operating characteristic curves was conducted with the Delong’s method. Univariable logistic regression model were used to evaluate the efficacy of each score on the main outcomes. All tests were two-sided and alpha error was set at 0.05. The statistical analysis was conducted in R version 4.1.2 [The R Project for Statistical Computing, Vienna, Austria (https://www.R-project.org/)].

## RESULTS

We have extracted data for 225 consecutive patients, which were classified as ‘TAAAD surgery’ in our institutional database over a period of 7 years (January 2014–March 2021). Of these, we have excluded 18 patients: 14 were not aortic dissection, 1 was a type B aortic dissection, 1 died before the operation, 1 was a duplicate and 1 had missing data on the main variables of interest (CT scan was missing). The final sample consisted of 207 patients who underwent surgical repair of TAAAD. The median age was 61.47 (IQR 52.12–71.48) years, and 140 patients were male (67.6%). The median ‘Radiological’ GERAADA score was 16.5 (IQR 11.9–24.05)% and the median ‘Clinical’ GERAADA score was 15.5 (11.9–24.5)%. Table 1 describes the GERAADA score characteristics for our population. The median EuroSCORE II was 6.21 (4.2–10.73)% and its characteristics are described in Table 2; 136 patients (65.7%) were in Penn class A, 48 (23.2%) in class B, 10 (4.9%) in class C and 13 (6.3%) were in class BC. Table 3 describes the operative characteristics.

**Table 1:** German Registry of Acute Aortic Dissection Type A score characteristics

Variables	Number of patients (%)
Age (years)	61.5 (52.1–71.5)
Gender (female)	67 (32%)
Resuscitation before surgery	6 (3%)
Previous cardiac surgery	2 (1%)
Intubation/ventilation at referral	9 (4%)
Cathecolamines at referral	22 (11%)
Aortic valve regurgitation	
No	106 (51%)
I–II	64 (31%)
III–IV	37 (18%)
Malperfusion (clinical and radiological criteria)	
None	150 (72%)
Coronary	15 (7%)
Visceral	18 (9%)
Peripheral	34 (16%)
Malperfusion (only radiological criteria)	
None	113 (55%)
Coronary	23 (11%)
Visceral	34 (16%)
Peripheral	65 (31%)
Preoperative hemiparesis	7 (3%)
Extension of dissection	
Aortic arch	180 (87%)
Supra-aortic vessels	109 (53%)
Descending or further downstream	136 (66%)
Location of primary entry in aortic arch	13 (6%)

Data are presented as median (interquartile range) or *n* (%).

**Table 2:** European System for Cardiac Operative Risk Evaluation II characteristics

Variable	Number of patients (%)	Variable	Number of patients (%)
Age (years)	61.5 (52.1–71.5)	Left ventricle ejection fraction	
Gender (female)	67 (32%)	Good (>50%)	179 (86%)
Renal impairment (CC)		Moderate (31–50)	25 (12%)
Normal (>85 ml/min)	86 (42%)	Poor (21–30)	2 (1%)
Moderate (50–85)	81 (39%)	Very poor (<20)	1 (0%)
Severe (<50)	37 (18%)	Recent MI	21 (10%)
Dialysis	1 (0%)	Pulmonary hypertension	
Extracardiac arteriopathy	47 (22%)	Moderate (31–55 mmHg)	2 (1%)
Poor mobility	3 (1%)	Severe (>55)	0 (0%)
Previous cardiac surgery	2 (1%)	Urgency	
Chronic lung disease	18 (9%)	Elective	0 (0%)
Active endocarditis	0 (0%)	Urgent	8 (4%)
Critical preop state	25 (12%)	Emergency	189 (91%)
Diabetic on insulin	1 (0%)	Salvage	10 (5%)
NYHA		Weight of the operation	
I	113 (55%)	Isolated CABG	0 (0%)
II	59 (28%)	Single non CABG	84 (41%)
III	20 (10%)	2 procedures	61 (29%)
IV	15 (7%)	3 procedures	62 (30%)
CCS class IV	0 (0%)	Surgery on thoracic aorta	207 (100%)

Data are presented as median (IQR) or *n* (%).

CABG: coronary artery bypass grafting; CC: creatinine clearance; CCS: Canadian Cardiovascular Society; IQR: interquartile range; MI: myocardial infarction; NYHA: New York Heart Association.

**Table 3:** Operative characteristics

Variables	Number of patients (%)
Cannulation site	
Ascending aorta	11 (5%)
Femoral	64 (31%)
Axillary	132 (64%)
CPB time, median (IQR)	178 (79)
Aortic clamping time, median (IQR)	88 (45)
Circulatory arrest time, median (IQR)	19 (30)
Neuroprotection	
DHCA	49 (29%)
ACP	118 (70%)
RCP	2 (1%)
Temperature (°C), mean (SD)	22.3 (4.7)
Aortic procedure:	
Patients with ascending aorta ± hemiarch replacement	207 (100%)
+ AVR	78 (38%)
Of which, Bentall operation	47 (23%)
+ Total arch replacement	26 (13%)
Of which, FET	12 (6%)
+ CABG	21 (10%)

Data are reported as median (IQR) or *n* (%).

ACP: anterograde cerebral perfusion; AVR: aortic valve replacement; CABG: coronary artery bypass grafting; CPB: cardiopulmonary bypass; DHCA: deep hypothermic cardiocirculatory arrest; FET: frozen elephant trunk; IQR: interquartile range; RCP: retrograde cerebral perfusion; SD: standard deviation.

### Postoperative outcomes

The 30-day mortality rate was 14.9% (31 patients), while overall in-hospital mortality was 16% (33 patients): of these patients, 10 died intraoperatively. Table 4 describes the postoperative outcomes and complications.

**Table 4:** Postoperative outcomes

Variables	Number of patients (%)
30-Day mortality	31(15%)
Mortality at discharge	33 (16%)
Death in theatre	10 (5%)
Death during postop recovery	23 (11%)
ICU length of stay (days), median (IQR)	10 (4–13)
Multiorgan failure	15 (7.2%)
Myocardial infarction	5 (2.4%)
Prolonged intubation (>48 h)	66 (32%)
Respiratory failure	26 (13%)
Return to theatre for bleeding	24 (12%)
GI complications	5 (2.4%)
Peripheral vascular complications	24 (12%)
Sternal wound infection	10 (4.8%)
Atrial fibrillation	53 (26%)
Permanent pacemaker implantation	5 (2.4%)
Stroke	38 (18%)
Peak creatinine (μmol/l), median (IQR)	136 (181.5)
Haemofiltration	42 (20%)

Data are reported as median (IQR) or *n* (%).

GI: gastrointestinal; ICU: intensive care unit; IQR: interquartile range.

### ROC curves for 30-day mortality

**Figure 2:**
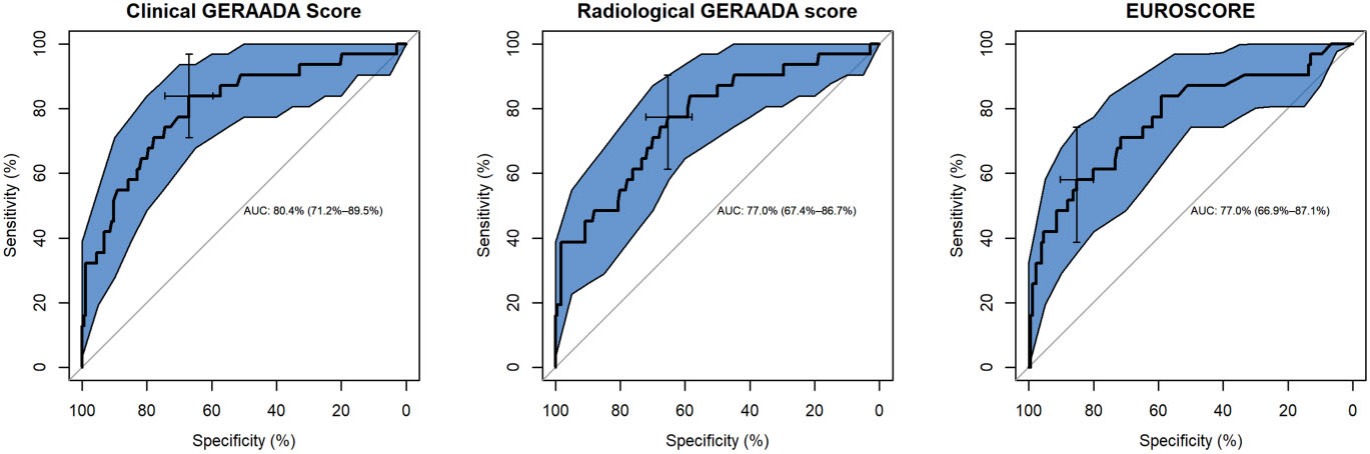
Comparison of the areas under the receiver operative characteristic curves of the German Registry of Acute Aortic Dissection Type A score (clinical), the German Registry of Acute Aortic Dissection Type A score (radiological) and the European System for Cardiac Operative Risk Evaluation II to predict 30-day mortality after type A acute aortic dissection surgery. AUC: area under the curve.

The Clinical GERAADA score had an excellent predicting ability for 30-day mortality [AUC 0.8; 95% confidence interval (CI) 0.71–0.89]. The calibration analysis for this model showed a Brier Score of 0.10 with Spiegelhalter’s *Z*-test = 0.13 (*P*-value = 0.89) and Hosmer and Lemeshow goodness-of-fit test 4.89 (*P*-value = 0.77), while the Radiological GERAADA score (AUC 0.77; 95% CI 0.67–0.87) was significantly less effective in predicting this outcome (*P*-value = 0.02 using Venkatraman methods). The calibration analysis for this model showed Brier Score of 0.09 with Spiegelhalter’s *Z*-test = 0.05 (*P*-value = 0.99) and Hosmer and Lemeshow goodness-of-fit test 3.67 (*P*-value = 0.89) The optimal cut-off point for the Clinical GERAADA score was 16.9 with a sensitivity of 0.84 and a specificity of 0.67. For the Radiological GERAADA score, the ideal cut-off point was 18.5 with a sensitivity of 0.77 and a specificity of 0.66. A univariable logistic regression model showed a significant role of the Clinical GERAADA score (OR 1.02; 95% CI 1.01–1.02, *p*-value < 0.01) and the Radiological GERAADA score (OR 1.11; 95% CI 1.06–1.17, *p*-value < 0.01) in predicting 30-day mortality. EuroSCORE underperformed Clinical GERAADA score with an AUC of 0.77 (95% CI 0.67–0.87) and a univariable logistic regression model for EuroSCORE II demonstrated a OR of 1.02 (95% CI 1.01–1.02, *P* < 0.01). The calibration analysis for this model showed Brier Score of 0.10 with Spiegelhalter’s *Z*-test = –0.009 (*P*-value = 0.99) and Hosmer and Lemeshow goodness-of-fit test 5.61 (*P*-value = 0.69) (Fig. 2).

### ROC curves for in hospital mortality

The Clinical GERAADA score had also an acceptable predicting ability for in-hospital mortality; although less effective than on 30-day mortality (AUC 0.77; 95% CI 0.67–0.87), the ideal cut-off point was again 16.9 with a sensitivity of 0.79 and a specificity of 0.67. The calibration analysis for this model showed Brier Score of 0.1 with Spiegelhalter’s *Z*-test = 0.04 (*P*-value = 0.98) and Hosmer and Lemeshow goodness-of-fit test 2.89 (*P*-value = 0.94). The Radiological GERAADA score (AUC 0.75; 95% CI 0.65–0.84) was less effective on this outcome and had an ideal cut-off point at 16.9, with a sensitivity of 0.82 and a specificity of 0.59. The calibration analysis for this model showed Brier Score of 0.11 with Spiegelhalter’s *Z*-test = –0.02 (*P*-value = 0.98) and Hosmer and Lemeshow goodness-of-fit test 7.43 (*P*-value = 0.49). EuroSCORE II also underperformed in predicting in-hospital mortality with an AUC of 0.75 (95% CI 0.65–0.85). The calibration analysis for this model showed Brier Score of 0.11 with Spiegelhalter’s *Z*-test = –0.02 (*P*-value = 0.98) and Hosmer and Lemeshow goodness-of-fit test 6.36 (*P*-value = 0.61).

### Subgroup analyses

Table 5 reports the performance of the Clinical and Radiological GERAADA and the EuroSCORE II in predicting mortality in specific groups of high-risk patients. The Clinical GERAADA score was the best predictor for most of these subgroups. The highest Clinical GERAADA scores were calculated for the following subgroups: intubation before surgery [predicted 59.04 (15.65)% vs actual 77.77%], inotropes before surgery [predicted 39.46 (20.22)% vs 59.09% actual], cardiogenic shock before surgery [predicted 37.79 (20.23)% vs 54.16% actual].

**Table 5:** Subgroup analyses in high-risk patients

Variables	Number of patients	30-Day mortality (%)	Clinical GERAADA predicted mortality	Radiological GERAADA predicted mortality	EuroSCORE predicted mortality
Age >65	92	18.68	19.98 (10.36)	21.63 (11.51)	12.01 (11.52)
Age ≤65	115	12.17	16.71 (12.01)	18.36 (13.06)	7.81 (7.70)
Female gender	67	11.94	15.66 (9.29)	17.13 (9.87)	8.19 (7.76)
Male gender	140	16.42	19.33 (12.10)	21.04 (13.36)	10.35 (10.54)
Inotropes before surgery	22	59.09	39.46 (20.22)	41.60 (21.17)	30 (15.62)
Intubated before surgery	9	77.77	59.04 (15.65)	60.56 (17.40)	35.35 (16.11)
Cardiogenic shock before surgery	24	54.16	37.79 (20.23)	39.87 (21.12)	29.21 (15.59)
Effusion/tamponade before surgery	84	22.62	21.46 (15.61)	22.87 (16.53)	13.84 (13.37)
Reduced LVEF	28	32.14	24.22 (18.08)	26.19 (19.06)	17.40 (14.36)

Data are reported as mean (SD).

EuroSCORE: European System for Cardiac Operative Risk Evaluation; GERAADA: German Registry of Acute Aortic Dissection Type A; LVEF: left ventricular ejection fraction; SD: standard deviation.

## DISCUSSION

In this study, we have confirmed the reliability of the GERAADA score in predicting 30-day mortality as previously reported by other centres [11] and we have also been able to discriminate a better predictive capacity of the ‘clinical’ GERAADA score compared to the ‘radiological’ GERAADA score. The GERAADA score was initially developed by Czerny *et al*. [3] to predict the postoperative mortality in these high-risk patients regarding the preoperative clinical status and was intended to serve as a useful instrument to foresee possible negative postoperative outcomes. Since its introduction, other studies have investigated the efficacy of the GERAADA score in TAAAD surgery with contrasting results: in a recent single-centre analysis [13], the authors found that this score showed a low predictive ability with an AUC of only 0.55. Sugiyama *et al*. [18] have also compared the GERAADA predicted mortality and found that the score tends to overestimate the actual mortality (14.3% predicted vs 6% actual): in this study, the authors were unable to calculate the accurate AUC due to a very low mortality rate. On the other hand, Luehr *et al*. [11] were able to find better reliability of the GERAADA score with an AUC of 0.67. In our study, we had a better result and by discriminating the clinical and radiological features of the GERAADA score, we have been able to demonstrate a very high discriminative power of the score with an AUC of 0.8 for the Clinical and of 0.77 for the Radiological version. This is an important finding as the GERAADA score does not precisely define the different types of malperfusion and although both scores were showing a good reliability, the Clinical GERAADA score, including both clinical and radiological findings, demonstrated a better predicting ability than the only radiological score. The main difference in how we calculated the Clinical and the Radiological GERAADA score relates the definition of coronary, visceral or peripheral malperfusion. Clinical history, physical examination, laboratory test and imaging are all used to diagnose malperfusion and the latest can be differentiated in ‘simple’ malperfusion and malperfusion syndrome. While the ‘simple’ malperfusion can be defined as the reduction or the absence of blood flow to an organ, the malperfusion syndrome is the late stage of malperfusion with organ and tissue damage [19, 20]. We suggest that the malperfusion syndrome may be underlined by the presence of clinical evidence, in addition to the radiological ones. Cho *et al*. compared outcome of patients operated for TAAAD without malperfusion and with preoperative evidence of subclinical malperfusion (defined as laboratory or imaging evidence of malperfusion) or clinical malperfusion, with associated signs and symptoms. They showed that the early and long-term mortality is not statistically different between the no malperfusion and the subclinical malperfusion groups, while clinical malperfusion significantly increases the risk for adverse outcomes [21]. The new Type, Entry, Malperfusion classification for the acute aortic dissections considers malperfusion differently if associated or not to clinical symptoms and we decided to apply the same principle to the GERAADA score [22]. It is evident that a better definition of the malperfusion criteria in the GERAADA score is needed as it could be fundamental to assess the real predictive capacity and capability of this score.

The Penn Classification, which is another simple tool to use in the setting of the TAAAD, has already shown good predictive ability in type A and type B dissection [9, 15] undergoing medical or surgical management; in our study, we decided to categorize the population according to the Penn Classification but not compare it with GERAADA score and EuroSCORE in view of the profound difference between a categorical classification and a numerical risk score. The Penn Classification is based on evidence of generalized and local malperfusion and ischaemia, and many authors have shown that patients in Penn class A (no malperfusion) have significantly better outcomes than patients in Penn class B, C or BC [9, 10, 23]. Malperfusion seems to be the fundamental risk factor for the outcomes after TAAAD surgical repair and the GERAADA score is currently the only quantitative short-term mortality scoring system that consider malperfusion as a variable. A better definition of the malperfusion criteria is needed to make its calculation unambiguous.

As in the recent study published by Nezić *et al.* [13], we have also compared the EuroSCORE II to the other scores, despite the fact that it was designed mainly for structural heart valve disease and/or coronary artery disease [24, 25] and it is therefore not specifically designed for TAAAD surgeries. In our analysis, the EuroSCORE had a good discriminative power but was inferior to the Clinical GERAADA score. Furthermore, many fundamental variables for the calculations of the EuroSCORE are usually not available in an acute setting and this could take to an inaccurate or not possible calculation of this. For these reasons, comparing to the EuroSCORE II, both the GERAADA score performs equally or better, are more specific for the setting of the TAAAD and are also easier to calculate as their variable need only a quick clinical assessment of the patient and a CT scan. Therefore, we think that there is no reason to use the EuroSCORE in the setting of the TAAAD and, as the Clinical GERAADA score performed better than all the other score, we recommend the use of this as new benchmark scoring system [9, 10, 15, 23].

### Limitations

This is a retrospective single-centre analysis, and the population is relatively small compared to the GERAADA registry. The CT scan evaluation has been performed by experienced surgeons and radiologists, but remain observer-biased, particularly for the assessment of the radiological malperfusion. Another limitation is that the definition of operative mortality of EuroSCORE II and GERAADA score are different: EuroSCORE is validated for predicting in-hospital mortality, while GERAADA score predicts 30-day mortality.

## CONCLUSION

The Clinical GERAADA score performs better than the other risk score, it is specifically designed for TAAAD and easy to calculate. Therefore, we strongly recommend it as the benchmark scoring system in this context. We have also defined new criteria for the assessment of the malperfusion and these need to be validated in further studies.

## Supplementary Material

ezad141_Supplementary_Data

## Data Availability

The data that support the findings of this study are available from the corresponding author (Vito D. Bruno) on request. The data are not publicly available due to restrictions because the information could compromise the privacy of research participants.

## Notes

Presented at the 36th EACTS Annual Meeting, Milano, Italy, 7 October 2022.

## ABBREVIATIONS

AUC: Area under the curve
CI: Confidence interval
EuroSCORE: European System for Cardiac Operative Risk Evaluation
GERAADA: German Registry of Acute Aortic Dissection Type A
IQR: Interquartile range
TAAAD: Type A acute aortic dissection
