# Supplementary material for: Validation of the German Registry for Acute Aortic Dissection Type A Score in predicting 30-day mortality after type A aortic dissection surgery
Source: Eur J Cardiothorac Surg. 2023 Apr 7;63(5):ezad141. doi: 10.1093/ejcts/ezad141 (PMC10824554; doi:10.1093/ejcts/ezad141)
Supplement: ezad141_Supplementary_Data [file ezad141_supplementary_data.docx]

Supplemental File


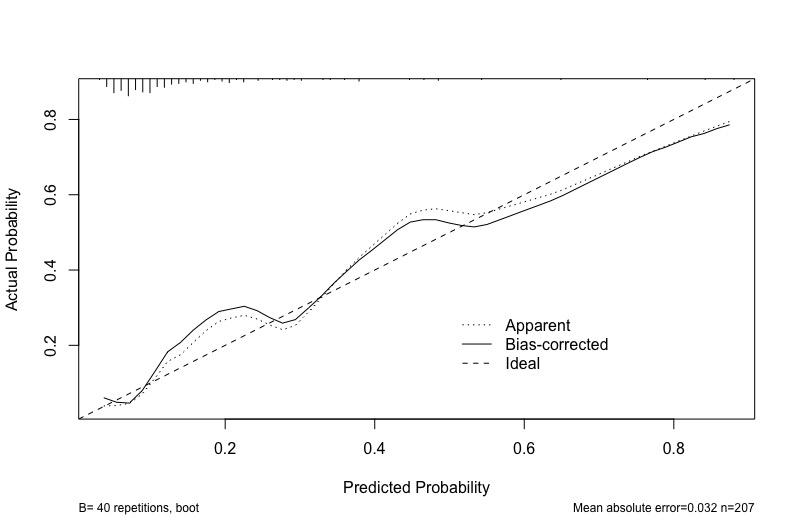


Fig. S1. Calibration plot for Clinical GERAADA score


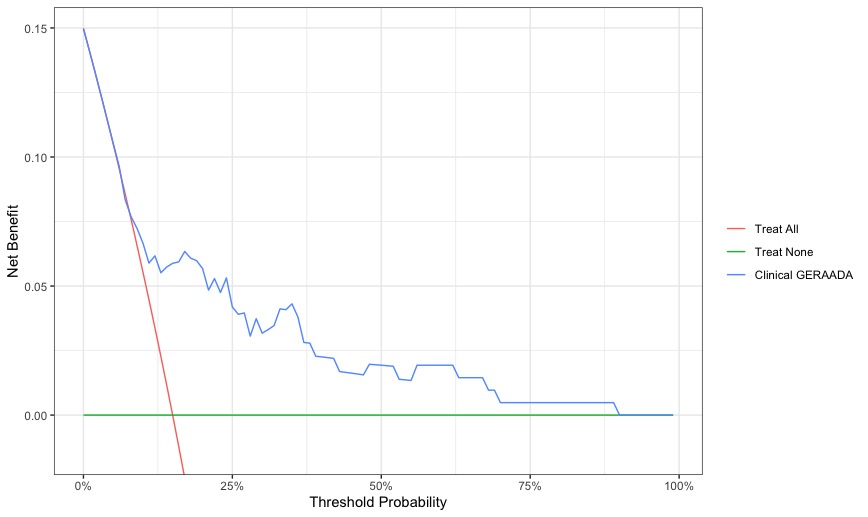


Fig. S2. Decision Curve Plot for Clinical GERAADA score


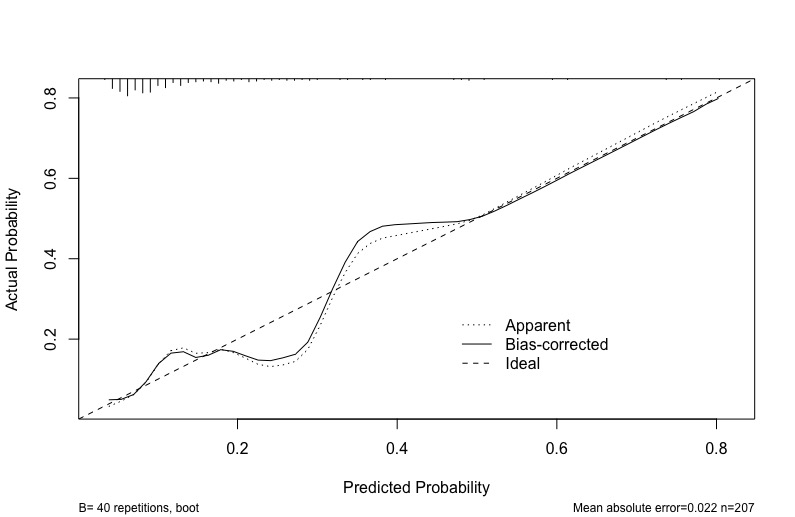


Fig. S3. Calibration Plot for Radiological GERAADA score


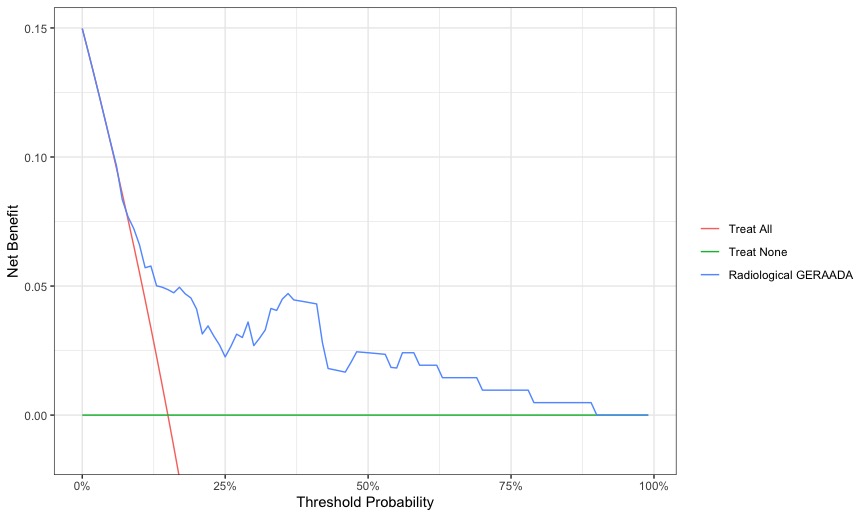


Fig. S4. Decision Curve Plot for Radiological GERAADA score


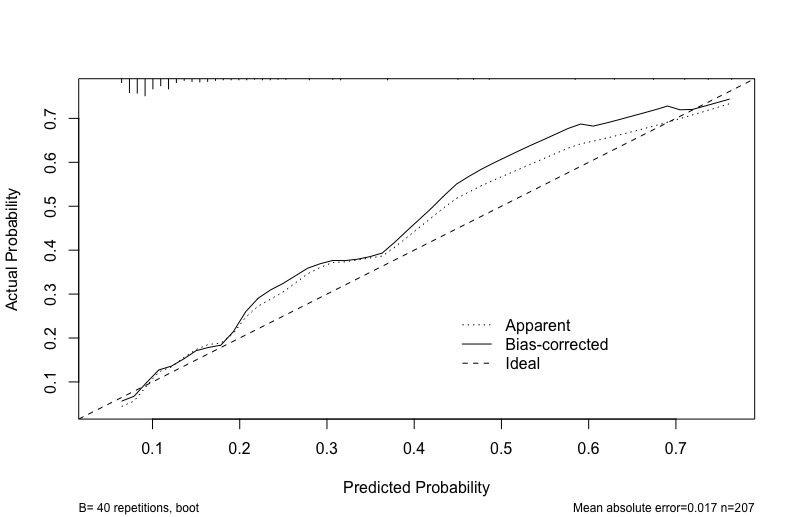


Fig.S5. Calibration Plot for Euroscore II


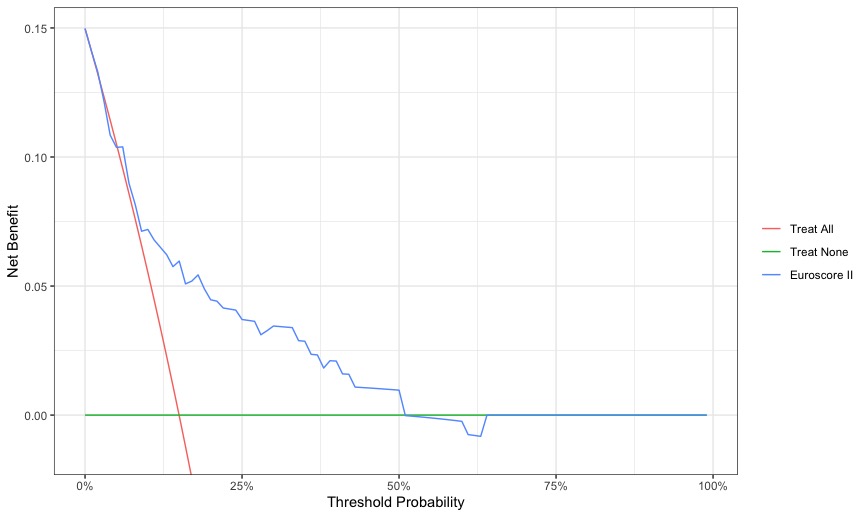


Fig. S6. Decision Curve Plot for Euroscore II
